# Hidradenitis suppurativa and psoriasis: the odd couple

**DOI:** 10.3389/fmed.2023.1208817

**Published:** 2023-07-07

**Authors:** Laura Macca, Federica Li Pomi, Ylenia Ingrasciotta, Pietro Morrone, Gianluca Trifirò, Claudio Guarneri

**Affiliations:** ^1^Department of Clinical and Experimental Medicine, Section of Dermatology, University of Messina, Messina, Italy; ^2^Department of Diagnostics and Public Health, University of Verona, Verona, Italy; ^3^Unit of Dermatology, Mariano Santo Hospital, Cosenza, Italy; ^4^Department of Biomedical and Dental Sciences and Morphofunctional Imaging, Section of Dermatology, University of Messina, Messina, Italy

**Keywords:** psoriasis, hidradenitis suppurativa, biologics, Th1, Th17, Th22, TNF-α, IL-12/23/17

## Abstract

Psoriasis and hidradenitis suppurativa are chronic inflammatory skin diseases that can develop together, negatively impacting on the patient’s quality of life. We aimed to review the most up-to-date information regarding the epidemiology, pathogenesis, clinical presentation and possible therapeutical choices in patients with both psoriasis and hidradenitis suppurativa, thus linking these two autoimmune and autoinflammatory conditions. A narrative review of articles dating from 2017 to 2022 has been performed using the PubMed database. We analyzed the case reports and case series found in the literature regarding patients who suffered from both psoriasis and HS. Psoriasis arose before hidradenitis suppurativa in the majority of cases, while only a minority of them had hidradenitis suppurativa before psoriasis. Interestingly, some patients suffered from paradoxical hidradenitis suppurativa following biological therapy administered to treat the already present psoriasis. Lastly, new biological drugs have been marketed with great success for the outcome of psoriasis, but similar progress did not happen for hidradenitis. Novel therapeutic approaches and lines of research are needed for the treatment of these pathologies, even if concomitant, in order to improve patient’s quality of life.

## 1. Introduction

Hidradenitis suppurativa (HS) is a debilitating, chronic, autoinflammatory cutaneous disease characterized by the inflammation of hair follicles in apocrine gland-rich areas (1, 2). Its prevalence estimates vary, ranging between 0.7 and 1.2%, with a more marked predisposition among females (3). It typically affects the intertriginous (axillary, submammary and inguinal), and anogenital areas, which later can lead to the development of abscesses, sinus tracts and scars, with devastating impact on patient’s quality of life (QoL) (1). The pathogenesis of HS is not yet well understood, but there is a general agreement on considering HS as a multifactorial disease having a plenty of implications with immunological factors and recruitment of self-perpetuating inflammatory mediators, which makes HS treatment a real challenge (4). HS is known to have a substantial impact on the QoL, being also associated with various comorbidities including increased cardiovascular risk, gastrointestinal, rheumatological and psychiatric comorbidities, as well as an increased risk of carcinogenesis (5). Having regard to this point of view, HS has been increasingly widely recognized as an inflammatory disease with several systemic implications, just like psoriasis (5). In fact, psoriasis is a chronic, immune-mediated, inflammatory disease which can also affect nails and joints (6). The reported prevalence is highly variable across countries ranging between 0.09 and 11.4%, with age-specific incidence rates showing a dual peak around 30–39 years of age and a second peak around 50–59 or 60–69 years of age (7, 8). Clinical presentation is morphologically and topographically heterogeneous. Plaque psoriasis, accounting for about 80% of all cases, is the classical phenotype, being characterized by well-defined, scaly, erythematous plaques, often covered by silvery scales, mostly affecting the extensor surfaces, scalp, and lumbosacral region (9). For a long time evaluated merely as a cutaneous disease, psoriasis is now recognized as a systemic inflammatory condition that shares pathogenetic pathways with several other disorders, including psoriatic arthritis, metabolic syndrome, cardiovascular diseases, inflammatory bowel diseases, uveitis, non-alcoholic fatty liver disease and HS (10). The coexistence of psoriasis and HS has been lately described, even if a possible association between them is yet to be firmly established. Several case reports of co-occurrence of HS and psoriasis suggest a causal relationship, but firm epidemiological data are lacking. To assess the link between HS and psoriasis, we performed a narrative review of the up-to-date literature on the topic (11–24).

## 2. Materials and methods

A bibliographic search was conducted on PubMed database^1^ using the search string: “Psoriasis” [All Fields] AND “Hidradenitis suppurativa” [All Fields]. Basing on the abstract content, we collected papers concerning this association. Only papers written in English language, concerning humans and with 5 years’ time limits were included. Papers identified as irrelevant to the topic in question were excluded.

## 3. Results

As of 1 November 2022, a PubMed search of “Psoriasis” AND “Hidradenitis suppurativa” yielded 21 articles. Among these, seven were excluded as not relevant and/or because the full text was not available, whereas 14 papers were selected as matching our search. Four case series and 10 case reports out of these 21 papers were studied and reviewed for epidemiological data, clinical features and treatment/outcomes. All these studies are summarized in Table 1.

**Table 1:** Reported cases of concomitant psoriasis and hidradenitis suppurativa.

Authors, reference number and year	Type of study	Patient no./Sex/Age (year)	Clinical course	Diseases severity	Previous treatment for HS	Previous treatments for psoriasis/PsA	Treatment for concomitant psoriasis/PsA and HS	Outcome
Yen et al. (24)	Case series	1/M/66 2/M/57 3/M/46	1/Psoriasis arising before HS 2/Psoriasis and PsA arising before HS 3/Psoriasis and PsA arising before HS	1/PASI 28.9; HS-PGA 4 2/PASI 23.4; HS-PGA 3 3/PASI 11.8; HS-PGA 3	1/Oral cefadroxil, topical gentamicin 2/Oral amoxicillin/clavulanate potassium 3/Oral cefadroxil	1/Acitretin 25 mg/day Methotrexate 7.5 mg/week Cyclosporine 200 mg/day NB-UVB phototherapy 2/Methotrexate 15 mg/week Sulfasalazine 1,000 mg/day Celecoxib 200 mg/day 3/Leflunomide 20 mg/day Acitretin 10 mg/day Cyclosporine 300 mg/day NB-UVB phototherapy	Adalimumab 80 mg on day, followed by 40 mg on day 8, and then every 2 weeks	3 months: 1/PASI 5.6; HS-PGA 0 2/PASI 1; HS-PGA 1 3/PASI 2.2; HS-PGA 1
Giuseppe et al. (23)	Case report	M/37	Psoriasis arising before HS	Both moderate	Oral rifampicin and clindamycin	Cyclosporine Infliximab 5 mg/kg	Secukinumab 300 mg in weeks 0, 1, 2, 3, 4 and then every 4 weeks	1 month: Regression of the abscesses and improvement of psoriatic lesions
Gadelha et al. (22)	Case report	F/22	PsAPASH and psoriasis of the scalp	Hurley stage III, Sartorius score 55	Clindamycin 300 mg every 8 h and metformin 500 mg twice daily	Capillary solution of salicylic acid and betamethasone dipropionate and antisecretory shampoo	Adalimumab 160 mg at week 0, 80 mg at week 2, 40 mg every week	2 months: Sartorius score 49
Tampouratzi et al. (21)	Case series	1/F/27 2/M/42	1/Psoriasis and PsA arising before HS 2/Psoriasis and PsA arising before HS	1/PASI 10.5, BSA 10%; Hurley stage II, IHS4 10 2/PASI 18.5, BSA 45%; Hurley stage II, IHS4 10	1/Topical steroids Methotrexate Apremilast 2/ Cyclosporine Methotrexate Golimumab Adalimumab Ustekinumam Secukinumab	1/Topical steroids Methotrexate Apremilast 2/ Cyclosporine Methotrexate Golimumab Adalimumab Ustekinumam Secukinumab	1/Certolizumab pegol 400 mg at week 0 and then every 2 weeks 2/Brodalumab 210 mg at week 0, 1, 2 and then every 2 weeks	3 months: 1/PASI 1, BSA 2%; IHS4 1 4 months: 2/PASI 1.5, BSA 8%; IHS4 3
Proietti et al. (20)	Case report	M/35	Psoriasis, HS, and recent history of SAMPUS	PASI 10.2; Hurley stage III, HS-PGA 3	Antibiotics and retinoids	Not reported	Apremilast 30 mg twice daily	3 months: PASI 2.5, HS-PGA 2
Lanna et al. (19)	Case report	M/55	HS arising before psoriasis	PASI 20, BSA 25%; Hurley stage II	Not reported	Not reported	Apremilast 30 mg twice daily	1 months: PASI 1; Hurley stage 0-I
Navarro-Triviño et al. (18)	Case report	M/58	Secukinumab-induced paradoxical HS arising in a patient with psoriasis and PsA	PASI 0; Hurley stage II	None (HS was a paradoxical reaction to Secukinumab)	Etanercept Adalimumab Secukinumab	Ustekinumab 45 mg	4 months: PASI 0; Hurley stage II
Vellaichamy et a (17).	Case report	F/27	Adalimumab-induced paradoxical HS arising in a patient with psoriasis and vitiligo	PASI 0; Hurley stage II-III	None	Topical treatments Apremilast Adalimumab	Adalimumab dosing increased to weekly and then stopped due to the onset of T-cell/histiocyte-rich large B-cell lymphoma (THRLBCL)	6 months: PASI 0; Hurley stage II-III
Yoshida et al. (16)	Case report	M/47	Psoriasis and HS	PASI 10.8; Hurley stage III, modified Sartorius score 102	Antibiotics and stab incision drainage	Topical steroids	Brodalumab 210 mg weekly for 2 weeks and then every 2 weeks	8 months: PASI 0; Hurley stage II, modified Sartorius score 30
Licata et al. (15)	Case report	M/50	Psoriasis arising before HS	PASI 25, BSA 20%; Hurley stage II	Cyclosporine 4 mg/kg/day Metotrexate 20 mg/week Adalimumab 40 mg/week	Cyclosporine 4 mg/kg/day Metotrexate 20 mg/week Adalimumab 40 mg/week	Risankizumab 150 mg at weeks 0, 4, and every 12 weeks thereafter	4 months: PASI 0, BSA 0; Hurley stage 0
Garcovich et al. (14)	Case series	1/M/73 2/M/51	1/Psoriasis, PsA and HS 2/Psoriasis, PsA and HS	1/PASI 5; DAPSA 55; Hurley stage III, HS-PGA 4 2/DAPSA 48.2; Hurley stage III, HS-PGA 5	1/Tetracyclines, rifampicin, and clyndamicin 2/Carbapenems, Cotrimaxozole Beta-lactams Cyclosporine Steroids Methotrexate Retinoids Infliximab Etanercept Adalimumab Secukinumab	1/Systemic steroids and NSAIDs 2/Carbapenems, cotrimaxozole, beta-lactams Cyclosporine Steroids Methotrexate Retinoids Infliximab Etanercept Adalimumab Secukinumab	1/Apremilast 30 mg twice daily 2/Apremilast 30 mg twice daily	4 months: 1/DAPSA 12.8; HS-PGA 2 2/DAPSA 16.7; HS-PGA 3
Gkini and Bewley (13)	Case report	F/19	Ustekinumab-induced paradoxical HS arising in a patient with psoriasis	PASI 13, Hurley stage III	Flucloxacillin, minocycline 100 mg/day, incisional drainage	Topical and conventional systemic drugs Ustekinumab 45 mg/3 months	Adalimumab 40 mg/week and surgery	6 months: Improvement (PASI and Hurley stage not reported)
Odorici et al. (12)	Case report	M/46	Secukinumab-induced paradoxical HS arising in a patient with psoriasis and PsA	PASI 12; Sartorius score 54	None (HS was a paradoxical reaction to Secukinumab)	Infliximab Etanercept. Adalimumab Secukinumab 300 mg/4 weeks	Ixekizumab 160 mg at week 0, 80 mg every other week for 12 weeks, and then 80 mg every 4 weeks	5 months: PASI: 2.2; Sartorius score 16
Musumeci et al. (11)	Case series	1/F/32 2/F/32	1/2/Adalimumab-induced paradoxical psoriasis arising in a patient with HS	1/PASI 5; Hurley stage III 2/PASI 7; Hurley stage II	1/2/Adalimumab 160 mg at week 0, 80 mg at week 2, 40 mg every week	1/2/None (Psoriasis was a paradoxical reaction to Adalimumab)	1/2Adalimumab discontinuation followed by treatment with topical steroids	3 months: 1/2/Improvement of psoriasis and no HS worsening (PASI and Hurley stage not reported)

## 4. Discussion

### 4.1. Epidemiology

The coexistence of psoriasis and HS had been revealed in several case reports and case series, whereas a established epidemiological evidence for an association between these diseases is still lacking. However, a large-scale population-based study revealed a significant association between psoriasis and HS, with an 80% increase in the odds for HS among patients with psoriasis versus matched controls (25). Interestingly, patients suffering from both diseases were younger and were more likely to be smokers and obese as compared to those affected exclusively by psoriasis, confirming the role of common inflammatory pathways in the pathogenesis of these conditions (25). Similarly, a recent nationwide Korean study evaluating the prevalence of psoriasis in HS-affected patients, highlighted that psoriasis was significantly increased compared to control subjects with a multivariate odd ratio (OR) of 4.6 (26). Moreover, HS was found to be associated to plaque psoriasis and palmar-plantar pustulosis in 9 and 11% of cases, respectively (27). The high prevalence of psoriasis in patients with HS was stated by the Authors on the basis of the common pathogenetic pathways (28).

### 4.2. Pathogenesis

The key moment in HS pathogenesis involves the infundibular epithelium of the apocrine glands and it is represented by the hyperkeratosis and the hyperplasia due to the interaction of environmental factors, genetic predisposition and immune dysregulation (5). This leads to follicular occlusion, followed by dilatation and rupture with a perifollicular lymphohistiocytic inflammation and development of retained hair tracts in pseudocysts and fistulas (29). Environmental factors include mechanical stress, metabolic syndrome, obesity, smoking, and dysbiosis (30). Genetic predisposition lays on alterations in Notch and γ-Secretase signaling pathways and in inflammasome response. As suggested by Moltrasio et al. three different forms of HS are to date recognized: the sporadic, the familial and the syndromic one (31). In each of these three forms the susceptibility and onset of the disease and the response to treatment are associated with different genetic variants, including genes encoding gamma-secretase subunits, while others involve autoinflammatory and/or keratinized genes (31). With regard to the immune dysregulation, several cytokines seem to be particularly expressed in the immunopathogenesis of HS (4). The “primum movens” is represented by the recognizing by Toll like receptors (TLRs) or NOD-like receptors (NLR) of bacteria and cellular debris as pathogen- and damage-associated molecular patterns (PAMPs and DAMPs) in the dermis, resulting in the activation of the innate immunity (30). PAMPs and DAMPs stimulate the inflammasome response thus activating the NOD-like receptor protein 3 (NLRP3) in macrophages and neutrophils followed by the production of caspase-1 and proinflammatory cytokines (e.g., TNF-α and IL-1β) (30). IL-1β causes the release of chemokines by fibroblasts, namely C-X-C motif chemokine ligand (CXCL)-1 and CXCL6, that will primarily draw neutrophils (30). Tumor necrosis factor-α (TNF-α), coming from macrophages and dendritic cells, upregulates TLR and provokes the releasing by keratinocytes of several chemokines, like CXCL8, CXCL11, C-C motif chemokine ligand (CCL)-20, and CCL2, which draw lymphocytes, neutrophils and monocytes into the dermis (30). Interleukin (IL)-23 and IL-12 produced by activated dendritic cells, favor polarization of CD4+ T lymphocytes toward T-helper lymphocytes type 17 (Th17) and Th1. On the same way, IL-17 produced by Th17 increases macrophage production of IL-1β and TNF-α, enhancing the immune response (32). Complement pathway activation by PAMPs and DAMPs takes part to the activation and recruitment of neutrophils performed by C3a and C5a (30). Interestingly, HS patients’ lesions express IL-10, an anti-inflammatory cytokine induced by the autocrine action of TNF in macrophages. IL-10 downregulates T cells and thus immune responses by suppressing the monocytes and macrophages production of pro-inflammatory mediators (33). Lastly, it has been noted a significant concentration of IL-36 α, −β, and -γ associated with a dysregulation on IL-36 receptor antagonist (IL-36RA) in lesional and perilesional HS skin (33). The unrestricted IL-36 signaling in these patients could serve as a potential target for future therapeutic strategies. Psoriasis shares several pathogenic pathways with HS, both being characterized by unbalanced interactions between the innate and the adaptive immune systems. Dendritic cells, activated by a variety of cell types (e.g., keratinocytes, natural killer T cells, macrophages, etc.), secrete IL-23 and IL-12, which in turn induce differentiation of native T-cells, respectively, to Th17 and Th1. IL-23 play a pivotal role in the survival and proliferation of Th17 and Th22 cells (9). Th17 cells produce IL-17 while Th1 and Th22 cells secrete TNF-α and IL-22, respectively. In psoriasis all these cytokines induce the activation of intracellular signal transduction in keratinocytes, leading to their overblown proliferation together with the augmented expression of angiogenic mediators, endothelial adhesion molecules, and immune cells infiltration into injured skin (34, 35). This immune dysregulation, based on genetic predisposition (presence of human leukocyte antigen (HLA) Cw6) and epigenetic modifications (desoxyribonucleic acid (DNA) methylation and histone acetylation), and environmental factors, such as smoking, alcohol, diet, infections, and mechanical stress, results in the inflammatory cascade that leads to erythematous scaly patches or plaques (34, 35). Epigenetic mechanism and their alterations play a pivotal role in keratinocytes differentiations and therefore in HS and psoriasis onset. Among them microRNAs (miRNA) are involved in keratinocytes proliferations and differentiations (36). MiR-146a and MiR-155have been found upregulated in psoriasis, thus promoting TNF expression, and correlating with IL-17 – driven inflammation (36).

### 4.3. Clinical manifestations

The typical lesions of HS include deep, inflammatory, painful nodules, abscesses, suppurative sinus tracts or tunnels, bridged scars and double-ended comedones (37, 38). Frequent complications of subcutaneous nodules and abscesses are represented by rupture, bleeding and production of purulent secretions, with serious consequences on the patient’s QoL. Patients are often restricted in their daily and interpersonal activities as the lesions are painful, foul-smelling, burning, and itchy (3). In a minority of patients, HS presents as “syndromic” being associated with other immune-mediated inflammatory diseases (39). The presence of HS is distinctive for the PASH (Pyoderma gangrenosum, Acne, and Suppurative Hidradenitis), PAPASH (Pyogenic Arthritis, Pyoderma gangrenosum, Acne, and Suppurative Hidradenitis), PsaPASH (Psoriatic Arthritis, Pyoderma gangrenosum, Acne, and Suppurative Hidradenitis) and PASS syndromes (Pyoderma gangrenosum, Acne, and Suppurative Hidradenitis, and Ankylosing Spondylitis) (39). The complex comorbidity profile and significant heterogeneity in clinical presentation, further complicates the diagnostic framing of these rare syndromic forms. Since clinical manifestations of HS are polymorphic, various phenotypic classifications have been proposed. With regard to this, Gonzalez-Manso has recently hypothesized two endotypic clusters. To cluster 1 belong non-obese males with early onset disease and a positive history of pilonidal sinus (40). Clinically, the lesions are mostly nodular, localized in the posterior sites; in addition, elevated serum levels of IL-10 and the presence of gamma-secretase mutations were found (40). Cluster 2 is mostly characterized by late-onset disease in obese females (40). Sinuses and abscesses, located in the anterior sites, are more frequent than nodules; furthermore, elevated serum levels of IL-1, C-reactive protein, IL-17 and IL-6 were found (40). Ultrasound imaging can further define lesion morphology and depth, thus enabling the precise staging of the disease (41). In this regard, a disagreement between clinical and ultrasound scores is emerging, as ultrasound is proving capable of detecting non-clinically evident HS lesions, in particular fistulas, suggesting the greater sensitivity of ultrasound compared to clinical scores (42). Similarly to psoriasis, numerous tools have been using. Hurley staging is the most recommended in the clinical setting due to its ease of use and usefulness (43). However, it is a static score since it does not calculate the number of injured areas and, above all, it does not allow to evaluate the activity of the disease or the response to treatment. A refined Hurley score was then proposed, that further distinguishes mild (A), moderate (B), and severe (C) forms in the context of stages I and II (44). The Modified Sartorius Score (MSS) is an additional scoring system which evaluates disease severity from a more clinically relevant point of view (32). A novel dynamic scoring system to assess HS severity is represented by International Hidradenitis Suppurativa Severity Score System (IHS4). The determination of IHS4 requires counting the nodules, abscesses and draining tunnels, making it clear to use in both research and clinical practice (45).

With regard to psoriasis, it usually presents with a plethora of clinical features that widely differ among some variants, including chronic plaque, guttate, erythrodermic, and pustular form (35, 46). Different variants can sometimes occur in the same patient (47). Beyond the peculiar characteristics, all phenotypes share peculiar features such as erythema, thickening and scales (48). Plaque psoriasis represents approximately 80 to 90% of all cases of psoriasis (49). It presents with sharply demarcated, erythematous, scaly patches or plaques and, although it can affect any part of the body, commonly occurs on the extensor surfaces, such as the elbows and knees, on the trunk, mostly in the sacral region, on the scalp and on the intergluteal fold (50). Based on these three characteristics and on the extent of the lesions, Psoriasis Area Severity Index (PASI) score and Body surface area (BSA) score are the most used tools to assess the severity of psoriasis and the treatment outcomes (51). The coexistence of HS and psoriasis in the same patient is rare, but well described in literature (11–24). In fact, it is well known that these two pathologies share common pathogenetic pathways which can induce both psoriatic plaques and the inflammatory nodules of HS.

On this topic, we reviewed all the case reports and case series available in the literature regarding patients who suffered from both psoriasis and HS. In most cases, psoriasis outbreak preceded the diagnosis of HS (12, 13, 15, 17, 18, 21, 23, 24). Only a minority of patients developed HS before psoriasis (11, 19). The analysis of data clearly suggests an important pathogenic link between the two entities, where the IL-23/Th17 axis plays a crucial role (15). Moreover, some patients suffered from paradoxical HS following biological therapy to treat the already present psoriasis (12, 13, 17, 18). Paradoxical immune-mediated inflammatory reactions, defined as the development of inflammatory immune-mediated tissue manifestations, are increasingly associated with biological targeted therapy (52). HS has also been reported to be paradoxically induced in patients treated with TNFα blockers, such as infliximab or adalimumab (53). Complete resolution of paradoxical hidradenitis suppurativa has been found to occur after drug discontinuation or switching to another biological agent, whereas reintroduction of the same biological agent resulted in the relapse of the disease (54). A case of unexpected flare of HS, probably due to a paradoxical reaction to ustekinumab, an anti-IL12/23 antibody, has been recently described (13). Also, secukinumab, a human IgG1κ monoclonal antibody that binds to the IL-17A, can trigger a paradoxical HS reaction (12). Marasca tried to summarize the immunological complex subtending the pathogenesis of autoinflammatory diseases, showing the potential dual role of secukinumab in two differen cases of HS, as trigger of pardoxical HS, successflully treated with the TNF-α inhibitor adalimumab, or capable of controlling HS caused by treatment with adalimumab, respectively (55). Th17/ILC3 lymphocytes, with high levels of IL-1β, TNF-α, IL-17 and peripheral recruitment of IL-17-producing neutrophils and Th17-cells represent the main hallmarks of hidradenitis suppurativa immune response pattern. In addition, paradoxical HS responses have been recently reported in patients with rheumatoid arthritis or psoriatic arthritis (PsA) during long-term treatment with TNF inhibitors, tocilizumab or rituximab (52–54). Physicians should be aware of this possible complication, so the causative agent should be stopped, and other treatment options be considered.

### 4.4. Therapies

**Figure 1:**
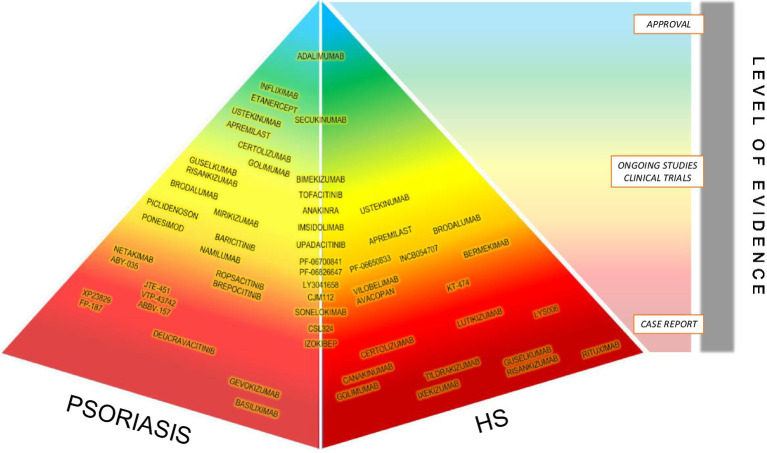
Approved and on-going studied biologic treatments for HS and psoriasis.

The hitch in the therapeutic management of HS is represented by the lack of approval to several biologic drugs by the United States Food and Drug Administration (FDA) and by the European Medicines Agency (EMA) (4). As recommended in international guidelines, the first ‘steps’ of oral treatment are represented by clindamycin and rifampicin, both administered at a dose of 300 mg twice daily for an average duration of 10 weeks in patients with Hurley stage II-III (30). The use of acitretin in HS, exerting antiproliferative activity in keratinocytes, is characterized by controversial outcomes given the high recurrence rates in monotherapy and overall response rates of 50% (30, 56). So, it is categorized as third line treatment (30). Isotretinoin has shown discordant results in HS, however it could be considered in patients with severe concomitant acne (30, 57, 58). The well-known influence of sexual hormones on the course of the disease, suggested by perimenstrual exacerbations and improvement during pregnancy, has been the rationale of use of estroprogestinic drugs (e.g., ethinylestradiol and noregestrel or cyproterone acetate, spironolactone, finasteride, metformin), either alone as monotherapy in mild/moderate disease or in combination therapy for more severe cases (30). The efficacy of systemic and intralesional steroids in acute HS flares has been widely demonstrated, even if high dose and protracted oral administrations of steroids are not recommended, given the possible flares after dose tapering, whereas the exact dosages and volumes to be used in intralesional administration remains undefined (30). For uncontrolled acute HS lesions, the surgical management may be a good therapeutic alternative, however clinical outcomes depend on the location of lesions: vulvar., perianal, and inferior breast excisions present higher recurrence rates. Variable results have been highlighted with laser and photodynamic therapy (PDT), however no standardized treatment protocols have been established, the variability of response is closely related to the used light source (30). The new frontiers of treatments are now represented by targeted biotechnological therapy with anti-TNFα and anti-IL-17 agents (30). Up to day, the unique biologic drug approved by the FDA and EMA for the treatment of moderate to severe HS is Adalimumab, a fully human monoclonal antibody targeting TNFα (59). Nevertheless, according with the real-life experience, its long-term efficacy seems to be highly variable (60). Infliximab, a chimeric mouse / human monoclonal IgG1 antibody against TNF-α, actually represents the second line therapy in moderate to severe HS unresponsive to adalimumab (33). Although several studies and anectodical reports have proved its efficacy and safety, it is not under investigation in any ongoing clinical trials (61, 62). Results about the efficacy of Golimumab, a human anti-TNFα monoclonal antibody, remain not encouraging and suggest that higher dosing may be needed for HS treatment (33). With regard to the last approved TNFα inhibitor, the humanized antibody IgG4 Certolizumab pegol, the effectiveness in HS across the published studies is promising, but, due to the still insufficient data, its role in HS warrants further investigation (33). About Canakinumab, a human monoclonal antibody against IL-1 β, no large clinical trials investigated its safety and efficacy in HS, in fact the results are not univocal and come from single case reports or case series (63–65). It also worth mentioning that Anakinra, a recombinant IL-1 receptor antagonist, might be a safe and effective treatment option for patients with acne inversa (33). Currently, the most promising biologics in phase III trials are anti-IL-17 antibodies, namely Secukinumab, a human monoclonal immunoglobulin G1 kappa antibody that binds to IL-17A, and Bimekizumab, a humanized IgG1κ monoclonal antibody that neutralizes both IL-17A and IL-17F (60). Furthermore, Bermekimab, a recombinant human IgG1 monoclonal antibody that binds IL-1α, is presently in phase II trials and shows encouraging results (60). Several research also investigated the efficacy of Brodalumab, a fully monoclonal antibody that binds to the IL-17 receptor A, and of Ixekizumab, a humanized IgG4 monoclonal antibody that neutralizes soluble IL-17A and IL-17 A/F. However, the few data on the use of Ixekizumab in HS come from case reports (12, 33) and only one ongoing early phase I clinical trial is trying to assess the response to treatment at a molecular level, through the collection of blood and tissue samples (66). Randomized controlled trials about the use of Ustekinumab, a IL-12/23 inhibitor, for patients with HS are lacking (33). IL-23 inhibitors (e.g., Guselkumab and Risankizumab), showed varied results as similarly complement C5a inhibitors (e.g., Vilobelimab, Avacopan), CD20 inhibitors (e.g., Rituximab), CD40 inhibitors (e.g., Iscalimab), phosphodiesterase-4 (PDE-4) inhibitors (e.g., Apremilast), anti-Il-36 agents (e.g., Spesolimab, Imsidolimab), leukotriene A4 (LTA4) inhibitor (e.g., LYS006), janus kinase (JAK) inhibitors (e.g., INCB054707, Tofacitinib, Upadacitinib) and CXC receptors (e.g., LY3041658) (33). Switching to psoriasis, based on the prevalence including about 125 million people in the world, it seems intuitive to understand why its therapeutic management enjoys a wide range of alternatives. In mild psoriasis, topical agents (e.g., steroids, vitamin D analogs, calcineurin inhibitors and keratolytics) remain the pivot of treatment together with the narrowband UV-B phototherapy (35). When conventional drugs (e.g., methotrexate, acitretin, cyclosporine, apremilast) are contraindicated or show primary or secondary ineffectiveness, the first line treatment for moderate to severe psoriasis is represented by biologics. Inhibitors of TNFα include Adalimumab, Etanercept, Certolizumab and Infliximab. Other biotech drugs block the p40 subunit of IL-12 and IL-23 (Ustekinumab), IL-17 (Secukinumab, Ixekizumab, Brodalumab and Bimekizumab), and the p19 subunit of IL-23 (Guselkumab, Tildrakizumab, Risankizumab and Mirikizumab) (35). When psoriasis occurs in association with HS therapeutic strategy should be chosen in concordance to diseases severity bearing in mind that almost all the biologics approved for the treatment of psoriasis are still off label for HS due to the absence of randomized controlled trials. In real life several treatments have been used for concomitant psoriasis/PsA and HS. Yen and Gadelha, in their studies described the improvement of both pathologies after the injection of Adalimumab and similarly Pistone referring to the use of Secukinumab (22–24). However, some cases reported in literature highlight paradoxical phenomena in which Adalimumab induced paradoxical HS in patients treated for psoriasis and vice versa (11, 17, 67, 68). Likewise, also Odorici and Navarro-Triviño pointed out paradoxical HS induced by Secukinumab used to treat psoriasis and PsA. Due to these reactions the drug was stopped and Ixekizumab and Ustekinumab were introduced respectively, with improvement of both conditions (12, 18). In non-responders, other biological drugs not yet approved by EMA and FDA, have shown encouraging results. Tampouratzi evidenced the efficacy of Certolizumab pegol and Brodalumab in two different patients previously treated with several drugs without response (21). Also, Yoshida conducted a study investigating the effectiveness of Brodalumab and Licata observed the complete resolution of the psoriatic and suppurative lesions 4 months after therapy with Risankizumab (15, 16). Lastly, a valid alternative option is represented by Apremilast as illustrated by Garcovic, Lanna, Proietti, although the results appear not always univocal (14, 19, 20). Approved and on-going studied biologic treatments for HS and psoriasis are summarized in Figure 1.

## 5. Conclusion and future perspectives

HS and psoriasis are chronic inflammatory skin diseases associated with a decrease in patients’ QoL, whose co-existence is increasingly being described in literature. It is assumed to depend on common pathogenic pathways in which IL-17 and IL-23 play a pivotal role. HS usually arises on a framework of already present psoriasis, and this can be explained by the different prevalence and incidences of the pathologies, being psoriasis much more frequent. However, the substantial difference that seem to emerge relates to the therapeutical approaches and their efficacy: while many biological drugs have been developed for psoriasis with excellent results in the reduction of the PASI and improving patients’ symptoms and QoL, the same goal was not reached for HS. Unlike psoriasis, the only registered biologic by the FDA and EMA in the treatment of moderate to severe HS is adalimumab, with sometimes unsatisfactory results. Nevertheless, the safety and efficacy of adalimumab have paved the way for comparing possible new drugs with adalimumab. Many other molecules with several immunological targets such as IL-12/23, IL-17, IL-23, IL-36, C5a, CD-20, CD-40, LTA4 and CXCR1/2 are currently under investigation for the treatment of moderate to severe HS. However, larger clinical trials of new therapeutic agents are mandatory in order to discover their effective therapeutic role, to evaluate the efficacy and safety and possible side effects.

## Author contributions

LM and FL: conceptualization, investigation, writing–original draft preparation, and project administration. GT and CG: methodology and supervision. LM, FL, and CG: validation. LM, FL, and YI: formal analysis and resources. CG: data curation and visualization. PM, GT, and CG: writing–review and editing. YI, GT, and CG: funding acquisition. All authors have read and agreed to the published version of the manuscript.

## Conflict of interest

CG has received consultation fees and/or grants for research projects, advisory panels and giving educational lectures from Wyeth-Pfizer, Abbott Immunology-Abbvie, Janssen-Cilag, Novartis, LEO-Pharma, LEO-Pharma Denmark, Ely-Lilly, Celgene, Merck-Serono, Sanofi-Aventis, Amgen and Almirall.

The remaining authors declare that the research was conducted in the absence of any commercial or financial relationships that could be construed as a potential conflict of interest.

## Publisher’s note

All claims expressed in this article are solely those of the authors and do not necessarily represent those of their affiliated organizations, or those of the publisher, the editors and the reviewers. Any product that may be evaluated in this article, or claim that may be made by its manufacturer, is not guaranteed or endorsed by the publisher.

## Footnotes

^1^https://ncbi.nlm.nih.gov/PubMed – accessed on 01 November 2022.
